# Emotional intelligence training among the healthcare workforce: a systematic review and meta-analysis

**DOI:** 10.3389/fpsyg.2024.1437035

**Published:** 2024-11-20

**Authors:** Chris Powell, Taylor Brown, Yang Yap, Karen Hallam, Marcel Takac, Tara Quinlivan, Sophia Xenos, Leila Karimi

**Affiliations:** Applied Health, School of Health and Biomedical Sciences, RMIT University, Melbourne, VIC, Australia

**Keywords:** emotional intelligence, EI, healthcare, burnout, systematic review, meta-analysis

## Abstract

**Background:**

The healthcare sector is acknowledged as a complex and challenging field. Increasingly, research highlights the importance of healthcare workers’ internal social and emotional skills in managing their well-being and enhancing their capacity to provide patient care and support to colleagues. Emotional Intelligence (EI) has been identified as a key factor in improving the health and performance of healthcare workers, leading to the implementation of numerous programs aimed at enhancing EI.

**Objective:**

This meta-analysis aims to evaluate the effectiveness of EI training interventions among healthcare workers, focusing on various intervention designs and their impact on EI improvement.

**Methods:**

The review encompassed 17 longitudinal studies, each implementing EI training interventions for healthcare workers aged 18 and over. The studies employed a variety of research designs.

**Results:**

All studies demonstrated an increase in EI following the intervention. However, methodological limitations within these studies might have led to an overestimation of the actual effects of the interventions.

**Conclusion:**

While the reviewed studies indicate a positive trend in EI enhancement post-intervention, the potential overestimation of effects due to methodological flaws necessitates caution. The findings underline the need for future research to explore the optimal duration and delivery methods for EI training in healthcare settings.

**Systematic review registration:**

The systematic review and meta-analysis have been pre-registered with PROSPERO [CRD42023393760]. Further details can be accessed at: https://www.crd.york.ac.uk/prospero/display_record.php?ID=CRD42023393760.

## 1 Introduction

The global healthcare sector is facing an escalating crisis in sustaining its workforce (Liu et al., 2017). This multifaceted challenge encompasses three key areas: (1) availability, referring to the existing pool of healthcare professionals; (2) distribution, focusing on the successful recruitment and retention of qualified staff; and (3) performance, relating to the productivity and quality of healthcare services provided (Liu et al., 2017). Numerous factors contribute to this complex issue. The healthcare environment is inherently challenging, marked by unique stressors such as demanding shift patterns, intense working conditions, and high workload pressures. Additionally, healthcare professionals often encounter workplace aggression (Mento et al., 2020), experience burnout, and face various psychosocial hazards, including job insecurity, workplace satisfaction issues, and the delicate balance between work and family life (Coutinho et al., 2018). Despite these hurdles, the effectiveness of patient-centred healthcare relies heavily on the collaborative efforts of healthcare personnel, who must work in unison with each other, the patients, and the broader health system (Santana et al., 2018).

The inherent occupational challenges impact patient care/experiences, healthcare team dynamics/efficiencies and workforce wellbeing. In relation to healthcare provision, the interpersonal behavior and approach of healthcare workers has shown to have a demonstrable direct impact on patient satisfaction, with kindness and empathy by physicians and nurses ranking only behind clinical outcomes as the most important predictor of global patient satisfaction (Schoenfelder et al., 2011). The pressures of these roles, combined with a range of psychosocial hazards discussed, can lead to occupational burnout (Dyrbye et al., 2017). Occupational burnout is associated with chronic exposure to work stress and is characterised by the qualitative dimensions of emotional exhaustion, cynicism, depersonalisation, and reduced sense of efficacy and meaning. Burnout also impacts patients as it worsens the quality of care, increases the risk of medical errors (Hall et al., 2016), and further reduces patient satisfaction and the therapeutic relationship (Panagioti et al., 2018).

More broadly, burnout among healthcare workers not only profoundly affects their health and well-being but also significantly impacts the functionality of healthcare systems at large. There has been a recent surge in focus on how burnout in healthcare providers correlates with diminished quality of patient care (Karimi et al., 2021). A growing corpus of primary research and systematic reviews has identified links between burnout and various critical aspects of healthcare delivery (Søvold et al., 2021). These include adherence to practice guidelines, communication effectiveness, the incidence of medical errors, patient outcomes, and safety metrics. Occupational burnout is directly linked to heightened occurrences of mental illnesses, psychological distress, trauma, and substance use disorders among healthcare professionals (Søvold et al., 2021; Dyrbye et al., 2019). From a systemic perspective, burnout contributes to increased staff turnover, higher rates of absenteeism, and presenteeism, which is characterized by reduced work performance (Dyrbye et al., 2019; Felton, 1998). These factors collectively lead to a deterioration in the overall quality of clinical care (Felton, 1998).

The potential risks to workers and patients have led to extensive research into factors that encourage positive interactions and effective coping among healthcare workers. A promising area of study is Emotional Intelligence (EI), which has been conceptualized in various ways to capture different facets of emotional and interpersonal skills. Mayer and Salovey (1997: p.10) ability-based model defined EI as “a set of interrelated skills concerning the ability to perceive accurately, appraise, and express emotion; the ability to access and/or generate feelings when they facilitate thought; the ability to understand emotion and emotional knowledge; and the ability to regulate emotions to promote emotional and intellectual growth.” Complementing this, Bar-On’s mixed model, as operationalized by the EQ-i, extends EI to include a broad spectrum of personality-linked traits, such as stress management and interpersonal skills, which are particularly relevant in the healthcare context (O'Connor et al., 2019; Tommasi et al., 2023; Sergi et al., 2021). Additionally, Trait EI, as introduced by Petrides and Furnham, explores self-perceived emotional abilities, such as resilience and adaptability, that align well with the psychological demands experienced by healthcare workers (O'Connor et al., 2019; Tommasi et al., 2023; Sergi et al., 2021). Research in the EI field has demonstrated that a specific set of interpersonal and intrapersonal skills and approaches have a positive impact on both healthcare workers (Vlachou et al., 2016) and the patients they treat (Karimi et al., 2021; O'Connor et al., 2019; Mattingly and Kraiger, 2019; Vlachou et al., 2016; Tawfik et al., 2019). While the prevalence of EI skills training in the healthcare sector is well-documented (Mattingly and Kraiger, 2019; Meyer et al., 2004; Baudry et al., 2018; Aldaod et al., 2019; Jiménez-Picón et al., 2021), the true scope and impact of such training on healthcare professionals remain largely unexplored.

EI training interventions, when effectively applied, have the potential to enhance adaptability, problem-solving skills, and coping strategies, which are critical for healthcare workers facing high-stress environments. Enhanced EI skills have been shown to build psychological resilience and promote improved emotional well-being. This association has some foundation in construct validity as the EI traits described initially by Goleman (2020), comprising empathy, self-awareness, social skills, self-regulation, and motivation, align with many of the characteristics that are impactful in the complex and challenging healthcare environment. There is a need to synthesise the extensive research on emotional intelligence, particularly focusing on the needs of healthcare workers. Recognising this, the present systematic review and meta-analysis aimed to assess the efficacy and workplace impact of EI skills training among healthcare workers.

## 2 Method

The review was conducted per the Preferred Reporting Items for Systematic Reviews and Meta-Analyses (PRISMA) checklist (Page et al., 2021) and the Synthesis Without Meta-analysis (SWiM) guidelines (Campbell et al., 2020) for reporting this study.

### 2.1 Study selection and eligibility

#### 2.1.1 Participants

Healthcare workers employed in a health setting, aged between 18 to 65 years old.

#### 2.1.2 Condition or domain

This study has focused on EI training intervention among healthcare workers. Only studies that used a validated measure of EI will be considered.

#### 2.1.3 Exposure of interest/intervention

EI training intervention.

#### 2.1.4 Comparator(s)/control

Control groups were present for randomised controlled trials, quasi-experimental control trials, and controlled before-after study designs. In non-randomised experimental groups with before-after scores were employed, treating baseline data as data for the “control” group.

#### 2.1.5 Outcomes

EI, measured as a dependent variable, was the primary outcome. All other meaningful outcome variables were considered as secondary outcomes (e.g., burnout, well-being, and mental health).

#### 2.1.6 Types of studies

Longitudinal design studies examining EI training interventions among healthcare workforces.

### 2.2 Search strategy

With the help of an experienced librarian, the systematic search was conducted from the 1st of January 1995 until the 31st of August 2022. A three-step search strategy was used.

1. An initial limited search of *MEDLINE* and *PsycINFO* were undertaken, followed by an analysis of text words contained in the title and abstract and of the index terms used to describe the article.
2. All identified keywords and index terms have been used to conduct a second search using the following databases: *MEDLINE, PsycINFO*, and *Embase*.
3. The reference lists of the retrieved articles and reports were hand-searched.

The following keywords have been used to search the databases with the article title, abstracts and body all searched: emotional intelligence, emotional quotient, EQ, EI, emotional competence; intervention, training, healthcare workers, healthcare, healthcare workforce, and longitudinal study.

#### 2.2.1 Inclusion criteria

The studies that met the following criteria were included in the systematic review:

1. Were written in English, used original data and were published in a peer-reviewed journal;
2. Were conducted among the healthcare workforce (minimum 10 workers per group);
3. Used EI training as an intervention;
4. Measured improvement in EI skills over time as an outcome; and
5. Had a longitudinal design, reporting pre and post-EI skills intervention assessments.

#### 2.2.2 Exclusion criteria

Studies that failed to meet the following criteria were excluded:

1. Only the abstract was available and/or a full-text version could not be located.
2. Secondary data, systematic review, grey literature, non-peer-reviewed publications, or conference abstracts.

### 2.3 Data collection and analysis

#### 2.3.1 Study selection

**Figure 1:**
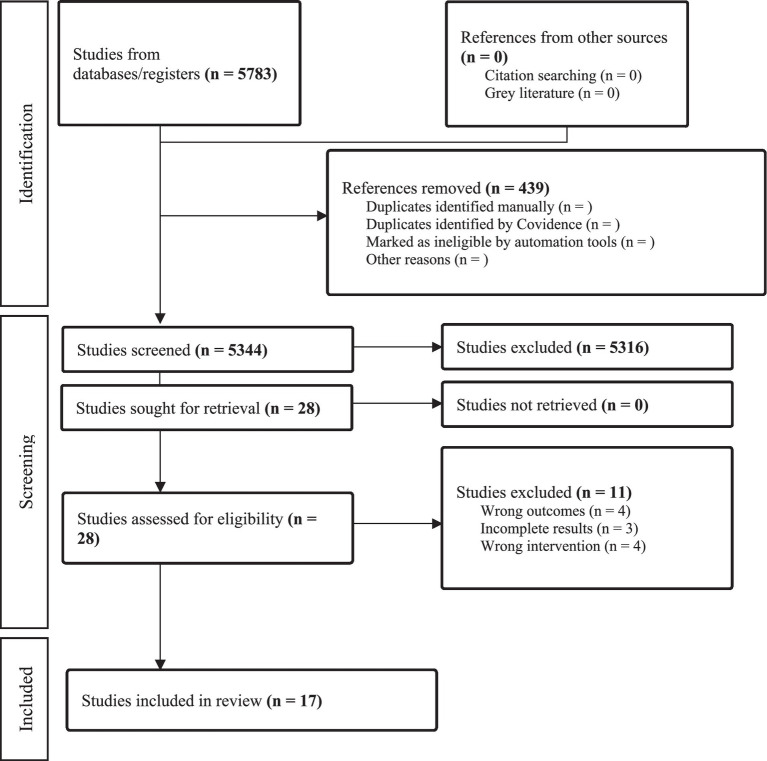
PRISMA chart: search results for EI training as an intervention.

Web-based systematic review software COVIDENCE was used for data selection and screening. All the searched studies were imported to Covidence. The duplicates were removed, and two independent reviewers (CP, YY) screened the references after creating the inclusion and exclusion criteria. The screening was performed in two steps: (a) the title and abstract screening and (b) full-text screening of the selected references. In both steps of the screening, the references were equally distributed between two reviewers, while a third independent reviewer (LK) was consulted when required. Reasons for the exclusion of full-text studies were recorded. The search results for article selection are presented in Figure 1.

#### 2.3.2 Data extraction and management

A template for data extraction was formed using the Synthesis Without Meta-analysis (SWiM) guidelines. Two reviewers (CP, YY) extracted and checked the data of the included studies. The information extracted from each study included:

1. Study characteristics (date of study, title, authors, and research question);
2. Methods (study design, primary outcome, secondary variables, exposure/s, potential confounders, and any other outcomes);
3. Participants’ demographics (country, age, sex, socioeconomic background, hours/week OR working arrangements, e.g., full-time, part-time, casual; and years of experience),
4. Outcomes (name and definition, how it was measured and reported), 5- control group,
5. Statistics (mean differences, their standard errors, *p*-values or confidence intervals). A second reviewer (CP/YY) cross-checked all extracted data.

A summary of the included articles is detailed in Table 1.

**Table 1:** Summary of characteristics of included studies.

Journal	Country	N	Age mean (M) and Standard deviation (SD)	Gender (%)	Sample population	Emotional Intelligence scale	Methodology
32	UK	60 Intervention = 33 Control = 35	Intervention M = 32 (6.16) Control M = 32.22 (7.1)	Female = 27 (45%) Male = 28 (48.3%)	First to third-year residents in the emergency medicine ward.	Sheering Emotional Intelligence Inventory	RCT
35	China	85 Intervention = 42 Control = 43	Intervention M = 38.11 (1.79) Control M = 40.04 (1.08)	Female = 50 (58.2%) Male = 35 (41.2%)	Psychology and nursing students engaging in an introductory psychology class.	Schutte Self-Report Emotional Intelligence Test (SSEIT)	RCT
33	Iran	135 Intervention = 62 Control = 73	32.11 (6.7)	Female = 73 (54%) Male = 62 (45.9%)	Nurses in units of Mohammad Vasei, Shahid Beheshti and Shahidan Mobini Hospitals in Sabzevar	Bar-On Emotional Quotient Inventory	RCT
37	The Netherlands	214 Intervention = 76 Control 1 = 71 Control 2 = 67	32.6 (9.2)	Female = 153 (71.5%) Male = 61 (28.5%)	Support staff from four Dutch residential treatment facilities for children, adolescents, and adults with moderate to borderline intellectual disabilities and challenging behaviors.	Bar-On Emotional Quotient Inventory	RCT
34	China	103 Intervention =53 Control = 50	Intervention M = 30.6 (5) Control M = 31.3 (6.6)		Medical and surgical wards	Wong and Law’s Emotional Intelligence Scale	RCT
36	Iran	52 Intervention = 25 Control = 27	Intervention M = 36.3 (6.7) Control M = 33 (6.3)		Intensive care nurses	Bar-On Emotional Quotient Inventory	RCT
38	Turkey	72 Intervention = 36 Control = 36	Intervention M = 18.83 (0.77) Control M = 19.2 (0.93)	Female = 57 (80.3%) Male = 14 (19.7%)	Freshmen nursing students at Adnan Menderes University	Bar-On Emotional Quotient Inventory	Quasi experimental with pre-test – post-test control groups
40	Australia	60 Intervention = 30 Control = 30	Intervention M = 18.83 (0.77) Control M = 19.2 (0.93)	Female = 57 (80.3) Male = 14 (19.7)	Registered nursing staff from eight units across two geographical sites within one health service in regional New South Wales, Australia.	GENOS Emotional Intelligence Self-Assessment	Quasi experimental with pre-test – post-test control groups
39	UK	70 Intervention = 34 Control = 36	Intervention M = 21.1 (2) Control M = 21.4 (2.4)	Female = 45 (62.3%) Male = 25 (35.7%)	Third year medical students	Bar-On Emotional Quotient Inventory	Quasi experimental with pre-test – post-test control groups
41	Australia	67 Intervention = 27 Control = 17			Staff and residents at two geographically separate residential care facilities in one organisation.	Bar-On Emotional Quotient Inventory	Controlled before-after studies
43	The Netherlands	60 Intervention = 34 Control = 26		Female = 44 (73%) Male = 16 (27%)	Staff members from two residential settings who supported people with mild to moderate behaviors and psychiatric problems.	Bar-On Emotional Quotient Inventory	Controlled before-after studies
42	Israel	31 Intervention = 16 Control = 15			Participants were from the hematology-oncology unit.	Bar-On Emotional Quotient Inventory	Controlled before-after studies
48	Spain	92	27.2 (11.23) years old	Female = 78 (84.8%) Male = 14 (15.2%)	Registered nurses and certified nursing assistants working with older adults.	Trait Meta-Mood Scale	Non-RCT
45	USA	117	24.01 (1.78) years old	Female = 64 (54.7%) Male = 53 (43.3%)	Dental students enrolled at The Ohio State University College of Dentistry.	Emotional Quotient Self-Assessment Checklist (EQSAC)	Non-RCT
46	Egypt	200	20.16 (0.37)	Female = 118 (59%) Male = 82 (41%)	Second-year nursing students.		Non-RCT
47	USA	31		Female = 22 (71%) Male = 9 (29%)	Medical residents	Bar-On Emotional Quotient Inventory	Non-RCT
44	USA	45	44.53 (9.96)	Female = 42 (93.3%) Male = 3 (6.7%)	Nurse managers from the healthcare system	Trait Emotional Intelligence Questionnaire-Short Form	Non-RCT

RCT is an abbreviation for randomized control trails. Additionally, some sections of the table are missing due to insufficient information provided by the authors and therefore, were not included.

#### 2.3.3 Risk of bias

The present study employed the adapted Cochrane Collaboration risk of bias assessment tool (Higgins et al., 2011), facilitated by the Covidence software, to rigorously evaluate the risk of bias in randomised trials. This tool appraises six distinct domains:

1. Unbiased participant distribution across study groups via random sequence generation and allocation concealment (selection bias);
2. Systematic differences between groups in the control and intervention group, addressed through participant and personnel blinding (performance bias);
3. Examination of outcome assessment blinding with consideration for potential prediction of intervention assignments (detection bias);
4. Addressing the systematic differences between the groups concerning participant withdrawals and information completeness pertaining to attrition and exclusions within the study (attrition bias);
5. Verifying the inclusion of all pre-specified outcomes in the study report (selective outcome reporting) and preventing the omission of outcomes based on their perceived significance or alignment with desired results (reporting bias);
6. Evaluating any unaddressed bias concerns, this broad domain encompasses factors such as disparities in heterogeneity and deviations from intended intervention as reported in the protocol (other biases).

Two independent reviewers (CP/YY) systematically employed the tool to assess each included study, documenting rationale and supporting details for risk of bias judgments in each domain (low, high, unsure). Discrepancies in bias judgments or reasoning were resolved through discussion between the two reviewers, and if needed, a third reviewer adjudicated as a mediator. Following the guidance outlined by Higgins et al. (2011), each domain was analysed, and a summary of bias judgments (low, high, unclear) was furnished.

### 2.4 Data analysis

The measurement of treatment effects was approached following the recent guidelines for systematic reviews that include both randomised and non-randomised studies (Higgins et al., 2023). For randomised controlled trials (RCTs), the analysis of continuous data employed standardised mean differences (SMD). SMDs were calculated when different instruments or scales were used to measure outcomes. This approach involved dividing the mean score difference between the intervention and control groups by an estimate of the pooled standard deviation, resulting in a ‘scale-free’ effect estimate. This ‘scale-free’ estimate allows for pooling across studies, regardless of the specific measurement scales employed in each study. Effect sizes were interpreted using common thresholds: SMDs less than 0.4 indicated a small effect, SMDs between 0.4 and 0.7 represented a moderate effect, and SMDs greater than 0.7 indicated a large effect (Cohen, 2013). Similarly, this procedure was repeated for studies using quasi-experimental control trials and controlled before-after study designs. In non-randomised experimental groups with before-after score SMD were employed, treating baseline data as data for the “control” group. When feasible, meta-analyses were conducted using MAJOR, a Meta-Analysis tool in Jamovi (Hamilton, 2018).

### 2.5 Assessment of heterogeneity

Given the diverse range of interventions, measures of EI, and follow-up durations in the included studies, our approach involves combining results from studies with similar designs. A random-effects model was employed to account for the anticipated heterogeneity among these studies. The degree of heterogeneity, represented as tau^2^, was calculated using the restricted maximum-likelihood estimator (Viechtbauer, 2005). Additionally, the assessment of heterogeneity includes the Q-test (Cochran, 1954) and the I^2^ statistic. If any level of heterogeneity is identified, signified by tau^2^ exceeding zero, an accompanying prediction interval for the actual outcomes is furnished.

## 3 Results

### 3.1 Study selection

The initial database search yielded a total of 5,783 studies. Following the removal of 439 duplicate records, 5,344 unique studies remained for thorough eligibility assessment. Upon conducting title and abstract screenings, a total of 5,316 studies were identified as irrelevant and subsequently excluded. The remaining 28 articles were independently assessed for eligibility by two reviewers based on the full-text review inclusion and exclusion guidelines. In alignment with these criteria, 11 studies were excluded due to including the wrong intervention of interest (*n =* 4), wrong outcomes (*n =* 4), or incomplete reporting of the results (*n =* 3). Consequently, the systematic review encompassed 17 studies for the final review. These studies included six randomised controlled trials (RCT) (Abbasi et al., 2018; Foji et al., 2020; Mao et al., 2021; Meng and Qi, 2018; Sharif et al., 2013; Zijlmans et al., 2015), three quasi-experimental with pre-test-post-test control designs (Erkayiran and Demirkiran, 2018; Fletcher et al., 2009; Kozlowski et al., 2018), three controlled before-after studies (Karimi et al., 2020; Tadmor et al., 2016; Zijlmans et al., 2011), and five non-randomised experimental designs (Non-RCT) (Frias et al., 2021; Partido and Stefanik, 2020; Ragab et al., 2021; Shahid et al., 2018; Sarabia-Cobo et al., 2017). The PRIMSA flowchart is presented in Figure 1.

### 3.2 Characteristics of included studies

The studies selected for this review span a publication timeline ranging from July 2009 to December 2021. Participants in the qualified studies were recruited from a range of settings. Twelve studies focused on healthcare settings (Abbasi et al., 2018; Foji et al., 2020; Mao et al., 2021; Sharif et al., 2013; Zijlmans et al., 2015; Kozlowski et al., 2018; Karimi et al., 2020; Tadmor et al., 2016; Zijlmans et al., 2011; Frias et al., 2021; Shahid et al., 2018; Sarabia-Cobo et al., 2017), whereas five studies centred around educational institutions (Meng and Qi, 2018; Erkayiran and Demirkiran, 2018; Fletcher et al., 2009; Partido and Stefanik, 2020; Ragab et al., 2021). Within healthcare settings, participants hailed from various medical domains, including hospital nursing and resident contexts (Foji et al., 2020; Mao et al., 2021; Zijlmans et al., 2015; Kozlowski et al., 2018; Frias et al., 2021), residential care facilities (Karimi et al., 2020; Zijlmans et al., 2011), nursing homes (Sarabia-Cobo et al., 2017), intensive care units (Sharif et al., 2013), hematology-oncology departments (Tadmor et al., 2016), emergency departments (Abbasi et al., 2018), paediatric settings (Sharif et al., 2013), and intense care units (Sharif et al., 2013). Further information concerning the characteristics of the included studies is provided in Table 1.

#### 3.2.1 Intervention

The included studies adopted diverse intervention methods to enhance EI among healthcare workers. Four studies focused on enhancing knowledge and understanding of EI (Abbasi et al., 2018; Foji et al., 2020; Sharif et al., 2013; Partido and Stefanik, 2020). This encompassed educational sessions focusing on learning and discussing EI (Foji et al., 2020; Sharif et al., 2013; Partido and Stefanik, 2020) and included educational tools such as pamphlets to further enhance knowledge (Abbasi et al., 2018). In contrast, one study implemented a persuasion intervention using a single, individualised text message (SMS) reminders (Kozlowski et al., 2018). Furthermore, five studies placed a significant emphasis on training to enhance EI skills (Zijlmans et al., 2015; Erkayiran and Demirkiran, 2018; Fletcher et al., 2009; Karimi et al., 2020; Zijlmans et al., 2011). These EI training programs took various forms, encompassing feedback mechanisms (Zijlmans et al., 2015; Zijlmans et al., 2011), group exercises (Erkayiran and Demirkiran, 2018; Fletcher et al., 2009), and skill development sessions (Karimi et al., 2020). Additionally, one study employed enablement techniques, incorporating two behavior change strategies (Frias et al., 2021). In this approach, an individualized action plan was created to integrate EI techniques into daily workflow. Furthermore, seven studies embraced a combination of two or more intervention types (Mao et al., 2021; Meng and Qi, 2018; Kozlowski et al., 2018; Tadmor et al., 2016; Shahid et al., 2018; Sarabia-Cobo et al., 2017). These interventions are summarised in Table 2.

**Table 2:** Summary of interventions across the studies.

Journal	Intervention description	Duration
RCT		
32	EI educational program that contained educational tools (e.g., pamphlets).	Comprised four sessions, conducted bi-weekly over two months.
35	The EI intervention included a general conference program and an in-group program. These programs focused on various aspects of emotional intelligence, with an emphasis on anxiety reduction and teaching participants how to manage stress and emotions.	Occurred across 10 sessions for two hours twice a week and included 4 sessions covering the general conference program, and 6 sessions for the in-group program.
33	EI intervention focused on group members’ referrals with each other, educating and discussing EI, and emotional regulation.	The training consisted of six sessions for two hours twice a week.
37	The training consisted of in-service training sessions on emotional intelligence and providing personalized feedback on EQ-I profiles. It also included video feedback coaching sessions.	The intervention occurred over two days and a follow-up was scheduled four months after the first training day.
34	The intervention explored one’s perception, awareness, and regulation of emotions to increase EI and resilience, decrease stress and improve the experience of patients seeking care.	The EI training consisted of two phases: an initial four-week theoretical training phase and an ongoing case discussion phase over 11 months.
36	The intervention aimed to educate on various aspects of EI, covering health, self-awareness, stress, its symptoms and management, the link between thoughts and emotions, emotional intelligence, emotion management, relationship skills, and self-management.	The intervention occurred over two days.
Quasi-experimental		
38	The teaching material was based on the Bar-On Emotional Quotient Inventory. The intervention covered theoretical information and interactive content and discussed interpersonal skills, coping with stress, and communication skills. Each session began with a warm-up activity and ended with an activity related to the content covered in the session.	The intervention consisted of 10 sessions of 60–75 min.
40	The intervention contained exercises designed to help participants to recognize and manage emotions in themselves and others.	The intervention group received a four-hour EI workshop, 30-min one-on-one coaching session, and individualized text message reminder.
39	The intervention was delivered and run through an external facilitator and consisted of individual and group exercises.	EI intervention was delivered over seven months with a total of seven EI sessions. The sessions were monthly and four hours long.
Controlled before-after studies		
41	EI intervention was based on the Personal Leadership Seminar framework. The intervention focused on three elements, including effective interactions with others; stress management; and actions supporting self-reflection, self-management, and self-motivation.	EI intervention occurred over six months.
43	The training consisted of in-service training sessions on emotional intelligence and providing personalized feedback on EQ-I profiles. It also included video feedback coaching sessions.	The intervention occurred over two days and a follow-up was scheduled 1.5 and 3.5 months after the first training day.
42	The intervention covered topics such as the definition and significance of EI in medical work, its impact on staff well-being, empathy, interpersonal skills, stress management, emotional awareness, impulse control, positive emotions, and optimism.	The intervention consisted of ten two-hour workshops spaced over two weeks apart.
Non-RCT		
48	The workshop focused on developing the four components of EI; perceiving emotions; using emotions to facilitate thinking; understanding emotions; and regulating emotions. In addition, each session incorporated techniques to improve skills like emotional regulation and understanding.	Consisted of four, four-hour sessions over four weeks. Each session was held at a one-week interval and included one hour of theory, two hours of group activities, and one hour of casework.
45	The EI program focused on three themes, including communication, ethics, and cultural humility.	A 10-week EI education program.
46	EI training incorporates two parts, the first phase includes seven sessions focused on education on EI and problem-solving. Part two included eight sessions and focused on practical skills for EI and problem-solving.	EI training incorporates two parts over two months. Part one included seven sessions and part two included eight sessions.
47	The EI training intervention focused on enhancing EI skills based on Goleman’s model. The first workshop addressed self-awareness and self-management, while the second focused on social awareness and social skills improvement.	The EI training intervention comprised two separate two-hour educational workshops, totaling four hours.
44	The EI training program consisted of a two-hour session teaching EI skills and creating individualized action plans, followed by monthly reminders over four months.	EI training contained a two-hour session and monthly reminders over four months.

#### 3.2.2 Duration of intervention

Furthermore, the durations of interventions in these studies exhibit noteworthy variations. Some interventions are relatively short-term, spanning just a few hours to a couple of days. For instance, Frias et al. (2021) delivered a concise two-hour training session, while Shahid et al. (2018) and Sharif et al. (2013). Conducted two separate two-hour workshops. In contrast, other interventions reviewed in the studies extended over several weeks to multiple months. For example, both Sarabia-Cobo et al. (2017) and Zijlmans et al. (2011, 2015) incorporated interventions potentially spanning 4 months, while Abbasi et al. (2018) structured the training program over two months with four sessions. A ten-session intervention appeared most popular, with four papers adopting this approach (Meng and Qi, 2018; Erkayiran and Demirkiran, 2018; Tadmor et al., 2016; Partido and Stefanik, 2020). Ragab et al. (2021) divided their intervention into two parts, with the first part comprising seven sessions and the second, eight sessions; however, they did not note the duration of these sessions. On the other hand, several interventions employed a long-term approach, extending over several months (Fletcher et al., 2009; Karimi et al., 2020). For further details on the characteristics of the included studies, refer to Tables 1, 2.

### 3.3 Risk of bias within studies

The assessment of internal validity employed the Cochrane Risk of Bias comparison tool (Higgins et al., 2011), which categorised the key criteria into high, low, or unclear risk. Two independent assessors (CP/YY) conducted an impartial evaluation of the identified citations. Most studies exhibited a high risk of selection bias. Specifically, approximately 53% were deemed to have a high risk, except for three studies, which were assessed as low risk (Foji et al., 2020; Mao et al., 2021; Erkayiran and Demirkiran, 2018) concerning random sequence generation. The remaining studies fell into the category of unsure risks, primarily attributed to a lack of adequate information regarding the employed randomisation methods, accounting for about 29% of the studies. This uncertainty also extended to attempts to conceal the allocation sequence, with eight studies identified as having a high risk (Zijlmans et al., 2015; Tadmor et al., 2016; Zijlmans et al., 2011; Frias et al., 2021; Partido and Stefanik, 2020; Ragab et al., 2021; Shahid et al., 2018; Sarabia-Cobo et al., 2017). The remaining articles were characterised as unsure risks (~53%), often due to insufficient information on the implementation of allocation sequencing measures and the study design used, such as pre-post designs, leading to inherent high bias risk.

Performance bias was assessed as high in nine of the studies (Meng and Qi, 2018; Sharif et al., 2013; Fletcher et al., 2009; Tadmor et al., 2016; Frias et al., 2021; Partido and Stefanik, 2020; Shahid et al., 2018; Sarabia-Cobo et al., 2017). Furthermore, a large proportion of the studies (~88%) were identified as having a high risk of detection bias. In contrast, attrition bias received a low-risk rating across all studies (~65%). The risk assessment ratings are available in Table 3 (with additional supporting judgment details in Supplementary Table S1).

**Table 3:** Risk assessment summary for the included studies.

Journal	Selection bias		Performance bias	Detection bias	Attrition bias	Reporting bias	Other bias
32	?	?	?	−	?	?	−
33	+	?	?	−	+	?	−
34	+	?	?	−	+	?	−
35	?	?	−	−	+	?	−
36	?	?	−	?	+	+	−
37	?	−	?	−	+	?	−
38	+	?	?	−	+	?	−
39	?	?	−	−	−	?	−
40	−	?	?	−	?	?	−
41	−	?	?	−	−	?	−
42	−	−	−	?	+	?	+
43	−	−	?	−	+	?	−
44	−	−	−	−	−	?	−
45	−	−	−	−	+	?	−
46	−	−	−	−	+	?	−
47	−	−	−	−	−	?	−
48	−	−	−	−	+	?	−
	Sequence generation	Allocation concealment	Blinding of participants and personnel	Blinding of outcome assessment	Incomplete outcome data	Selective reporting	Other sources of bias

The assessment of internal validity employed the Cochrane Risk of Bias comparison tool (Higgins et al., 2011), which categorized the key criteria into high (−), low (+), or unclear risk (?).

### 3.4 Effectiveness of EI interventions

The subsequent sections outline the efficacy of EI interventions tailored for healthcare professionals, and this analysis has been be categorised based on study design, encompassing randomised controlled trials (RCT), quasi-experimental studies with control groups, controlled before-after investigations, and non-randomised experimental designs (non-RCT). Out of the 17 studies encompassed within this review, seven studies (Zijlmans et al., 2015; Kozlowski et al., 2018; Karimi et al., 2020; Tadmor et al., 2016; Zijlmans et al., 2011; Ragab et al., 2021; Shahid et al., 2018) were excluded from the meta-analysis due to the unavailability of data, rendering re-analysis unfeasible.

#### 3.4.1 Results of individual studies

A review of several studies, categorised into different research methodologies, sheds light on the effectiveness of EI interventions within the healthcare workforce. Nearly all included studies in this review reported statistically significant increases in EI after participating in an EI-related intervention, except for one non-randomised experimental study (Frias et al., 2021), which reported an improvement that was not statistically significant. Five of these studies employed an RCT design (Abbasi et al., 2018; Foji et al., 2020; Mao et al., 2021; Meng and Qi, 2018; Sharif et al., 2013; Zijlmans et al., 2015). All these RCT studies reported statistically significant increases in EI following participation in educational and training EI interventions, with effect sizes varying between 0.51 to 3.8. When considering the effect size threshold as small (SMD < 0.4), moderate (0.4 to 0.7), and large (> 0.7), most studies had between-group effect sizes that were large (*n* = 3) for increasing participants EI, followed by medium (*n* = 2). The most substantial effect sizes were observed in Meng and Qi (2018) (SMD = 3.8), Abbasi et al. (2018) (SMD = 1.45), and Mao et al. (2021) (SMD = 1.28).

In addition to RCT studies, three studies employed a quasi-experimental design with control groups (Erkayiran and Demirkiran, 2018; Fletcher et al., 2009; Kozlowski et al., 2018). However, it is essential to note that due to data unavailability in Kozlowski et al. (2018) study, re-analysis was infeasible, rendering it unaccounted for in the meta-analysis. Yet, this study reported a significant increase in EI following a single EI training session, although the effect remains unclear due to limited findings. Both Erkayiran and Demirkiran (2018) and Fletcher et al. (2009) reported improvements in EI among medical students after the EI training programs, with the largest effect size observed in Erkayiran and Demirkiran (2018) (SMD = 0.93) and Fletcher et al. (2009) reporting a moderate effect (SMD = 0.53).

Three studies in this review adopted controlled before-after designs (Karimi et al., 2020; Tadmor et al., 2016; Zijlmans et al., 2011). These studies utilised Bar-On’s conceptualisation of EI and assessed EI levels through the Bar-On EQ-i scale (Bar-On, 1997). These studies were not included in the meta-analysis due to limited available data. However, a summary of their findings based on available information is provided in Table 4.

**Table 4:** Summary of the data reported within the studies.

First author (year)	Baseline EI M (SD)	End of Intervention EI M (SD)	Significance (*p*-value)	Results: secondary outcomes	Summary
RCT					
Abbasi et al. (2018)	I = 94.73 (13.2) C = 93.23 (11.1)	I = 116 (18.84) C = 93.4 (9.6)	*p* < 0.001		There was no significant difference in EI between the intervention and control groups before the intervention. But after the intervention, there was a significant difference. The findings showed that among the components, residents had the highest mean score for self-awareness and the lowest for social skills.
Meng and Qi (2018)	I = 21.27 (4.28) C = 21.32 (4.54)	I = 38.67 (4.11) C = 22.16 (4.48)	*p* < 0.001	The nursing students in the EI group reported decreased perceived stress and increased communication skills after the intervention, while the control group did not show much change.	The results showed that the EI intervention was associated with lower tension, higher verbal, audible and feedback skills, and increased perceived EI among the nursing students compared to the control group.
Foji et al. (2020)	I = 319.06 (34.02) C = 333.76 (31.62)	I = 350.11 (29.67) C = 330.49 (43.84)	*p* < 0.001	The results showed that EI training significantly improved the general health scores of the nurses in the experimental group. As the nurses’ EI scores increased, their general health scores decreased, indicating better health.	EI training was effective in promoting the general health of nurses by reducing anxiety, stress, and other health problems. The training helped the nurses develop skills to better manage their emotions and improve their well-being.
Zijlmans et al. (2015)			*p* < 0.001	Trained staff showed increased task-oriented coping and more positive emotions like confidence and relaxation. The training only had a limited impact on staff experiencing negative emotions.	Staff training programs focused on EI and interactions was effective in improving the EI and coping of staff working with clients with ID, though it had limited impact on staff experiencing negative emotions. The effects of the training appeared to persist for at least four months after the training ended.
Mao et al. (2021)	I = 54.67 (7.28) C = 58.24 (7.24)	I = 62.81 (7.21) C = 53.53 (7.18)	*p* < 0.001	The results showed that the EI training significantly Improved nurses’ resilience scores and reduced nurses’ perceived stress levels. Additionally, the intervention led to small but significant improvements in inpatients’ experience.	EI training can benefit nurses by improving their EI, resilience, and stress management, which in turn can enhance the care they provide to patients.
Sharif et al. (2013).	I = 319 (33.2) C = 324.7 (27.5)	I = 337.9 (33) C = 320.2 (23.4)	*p* < 0.001	EI can significantly improve general health, leading to lower general health scores after the training.	Providing EI training to ICU nurses was effective in improving their general health and well-being by increasing their EI.
Quasi-experimental					
Erkayiran and Demirkiran (2018)	I = 186.19 (34.54) C = 191.46 (30.24)	I = 233.53 (42.14) C = 199.4 (30.22)	*p* = 0.04	The interpersonal relationship style scores of the training group increased significantly after the training.	The study found that providing EI skills training to nursing students can significantly improve their EI and interpersonal relationship styles.
Kozlowski et al. (2018)	I = 3.77 (0.18) C = 4.16	I = 4.09 (0.25) C = 4.16	*p* < 0.001		The intervention group had a significant increase in their EI scores from pre-to post-training, while the control group did not show any significant changes.
Fletcher et al. (2009)	I = 95.9 (11.9) C = 98.8 (13.9)	I = 104 (10.1) C = 96.9 (15.8)	*p* < 0.001		The intervention group showed significantly higher increases in their EI scores compared to the control group, suggesting the training had a positive effect.
Controlled before-and-after					
Karimi et al. (2020)	I = 434\u00B0C = 433	I = 470\u00B0C = 430	*p* < 0.001	Those who received EI training showed higher self-reported quality of resident care and psychological empowerment, and improved general well-being.	EI training was effective in improving the job performance of aged care workers in Australia. The training helped develop their emotional skills which led to higher quality of resident care, better well-being, and psychological empowerment.
Zijlmans et al. (2011)		Mean difference I = 11.03 (7.99) C = 5.88 (6.02)	*p* < 0.001		The study found that a training program focusing on EI, combined with video feedback, was effective in improving the EI of staff members.
Tadmor et al. (2016)	Sample M = 97.9	I = 105.6	*p* < 0.001		The staff showed significant improvements in their total EI score and scores on all 5 major EI scales after the training.
Non-RCT					
Sarabia-Cobo et al. (2017)	22.79 (8.32)	28.2 (5.32)	*p* < 0.05	Moderate to strong correlations were found between the EI scores and different coping styles, indicating that higher EI is linked to more effective coping.	Workshops focused on EI and coping styles can significantly improve the EI and coping of nurses and nursing assistants working with older adults.
Partido and Stefanik (2020)	111.88 (9.7)	118.76 (11.17)	*p* < 0.001		EI training in the course was effective in improving dental students’ EI levels.
Ragab et al. (2021)	28.5% satisfactory knowledge of EI	89.5% satisfactory knowledge of EI	*p* < 0.001	After the training program, a significant improvement in their problem-solving skills was found, which continued at the follow-up phase.	EI training can significantly improve EI and problem-solving knowledge skills.
Shahid et al. (2018)	Median = 110	Median = 114	*p* = 0.04	After the intervention, significant increases in stress management and overall wellness scores were reported.	Teaching EI skills can be beneficial to increase EI and other related areas including improved stress management skills, promote wellness, and prevent burnout.
Frias et al. (2021)	5.77 (0.5)	5.97 (0.54)	*p* = 0.18	Transformational leadership characteristics increased after the training, but this was not significant. Participants scored higher on EI and transformational leadership characteristics compared to averages, and lower on passive-avoidant leadership characteristics.	While EI scores and transformational leadership characteristics increased after the training, the changes were not statistically significant.

Intervention is abbreviated as I and control is abbreviated as C.

In the case of non-RCT studies, three studies were included in the meta-review (Frias et al., 2021; Partido and Stefanik, 2020; Sarabia-Cobo et al., 2017). The SMD within these studies ranged from 0.06 to 0.65. Interestingly, all three studies concurred in reporting a noticeable increase in EI as a result of the intervention. However, it is essential to highlight that most of these studies indicated a small effect size (*n* = 2), besides the study conducted by Partido and Stefanik (2020) which was found to have a moderate effect size (SMD = 0.65).

In terms of secondary measures, EI-derived interventions generally increased various aspects both in individual and workplace contexts. The studies revealed positive outcomes, with reported enhancements in general health and stress management (Foji et al., 2020; Mao et al., 2021; Sharif et al., 2013; Karimi et al., 2020). Moreover, the interventions contributed to increased resilience (Mao et al., 2021) and empowerment (Karimi et al., 2020). Skill development was a prominent feature, with reported improvements in communication (Meng and Qi, 2018) and problem-solving abilities (Ragab et al., 2021). In workplace settings, the interventions were associated with an improved quality of care (Mao et al., 2021; Karimi et al., 2020) and the development of transformative leadership skills (Frias et al., 2021). For a more detailed breakdown of the study results, examine Table 4.

#### 3.4.2 Random effects model: effectiveness of EI intervention

**Figure 2:**
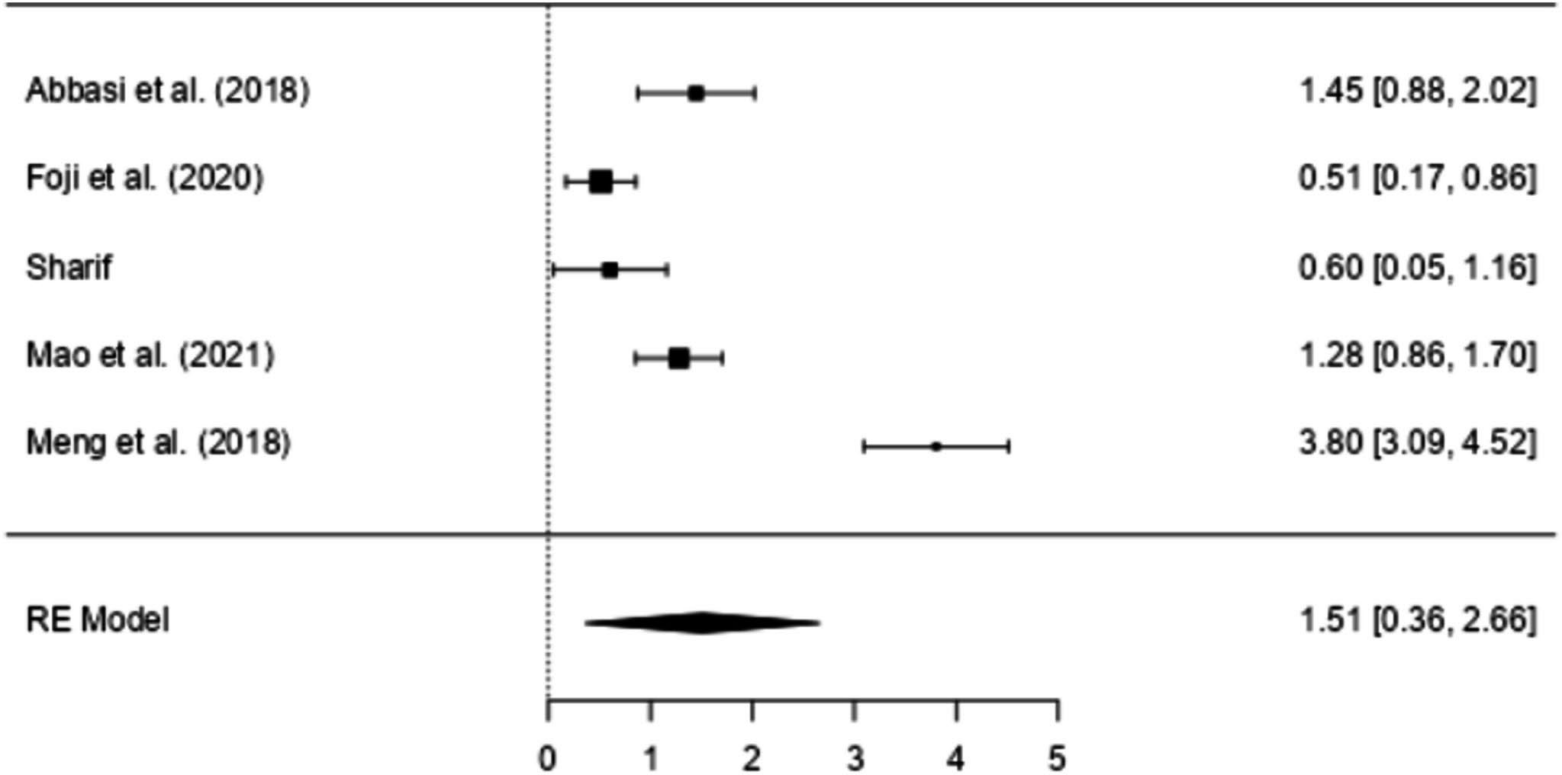
Forest plot of RCT assessing EI intervention effectiveness.

**Figure 3:**
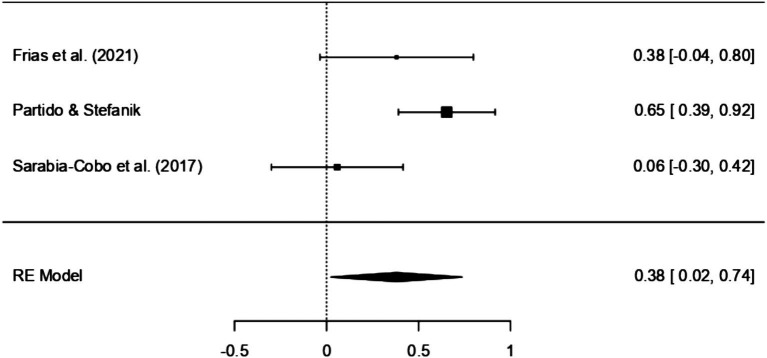
Forest plot of non-RCT assessing EI intervention effectiveness.

The results of the Random Effects Models are presented in Table 5. Within the RCT model, which incorporates five distinct studies, there appears to be a significant positive correlation between the interventions employed in the studies and the corresponding EI scores (SMD = 1.51, 95% CI [0.36, 2.66], *p* < 0.01). An examination of the 95% prediction interval for the actual outcomes reveals a range spanning from −1.25 to 4.27. Consequently, while the overall estimated outcome leans towards a positive effect, it is important to acknowledge that, in certain studies, the actual effect might, in fact, be negative. A similar pattern is observed in studies adopting Quasi-experimental designs (SMD = 0.71, 95% CI [0.32, 1.1], *p* < 0.001), suggesting a large effect according to Cohen’s criteria (Cohen, 2013). However, when non-RCT designs are employed, effect sizes diminish and reflect a lower impact (SMD = 0.38, 95% CI [0.02, 0.74], *p* < 0.05). Once again, the prediction interval for the actual outcome ranges from −0.24 to 1.01, implying that while the average effect is positive in some studies the true outcome may be negative. Further examination of the data distribution is permitted by Figures 2, 3, which display the effect size, and observed direction of the interventions on EI levels detected in each selected study.

**Table 5:** The results of the random effects model across the methodology categories.

		Effect size and 95% confidence interval				Prediction interval		Heterogeneity statistics					
Methodology category	Number of studies	Standard mean difference (SMD)	Standard error	Lower limit	Upper limit	Lower limit	Upper limit	Tau	Tau^2^	Q-value	df	*p*-value	I-square (%)
RCT	5	1.51	0.58	0.36	2.66	−1.25	4.27	1.28	1.64 (SE = 1.21)	71.55	4	*<* 0.001	96.31%
Quasi-experimental	2	0.71	0.19	0.32	1.1								
Non-RCT	3	0.38	0.18	0.02	0.74	−0.24	1.01	0.26	0.07 (SE = 0.09)	6.97	2	0.03	69.33%

Restricted maximum-likelihood is the selected Tau^2^ estimator. Please note that for quasi-experimental designs, the limited number of included studies (*n* = 2) precluded a formal heterogeneity test due to the small study size.

### 3.5 Heterogeneity

The results of the heterogeneity test are summarised in Table 5. In the realm of RCTs, our assessment of the five studies included in the meta-review unveiled substantial heterogeneity in their outcomes (Q = 71.55, *p* < 0.001). A similar observation was made for non-RCT designs, where our investigation of the three included studies indicated heterogeneity in their results (Q = 6.97, *p* < 0.05). This implies statistically significant differences between study outcomes, potentially stemming from variations in effects or study populations. However, it is important to acknowledge that, owing to the limited number of studies available for Quasi-experimental designs (*n* = 2), a formal heterogeneity test was not conducted in this case.

### 3.6 Reporting bias assessment

**Figure 4:**
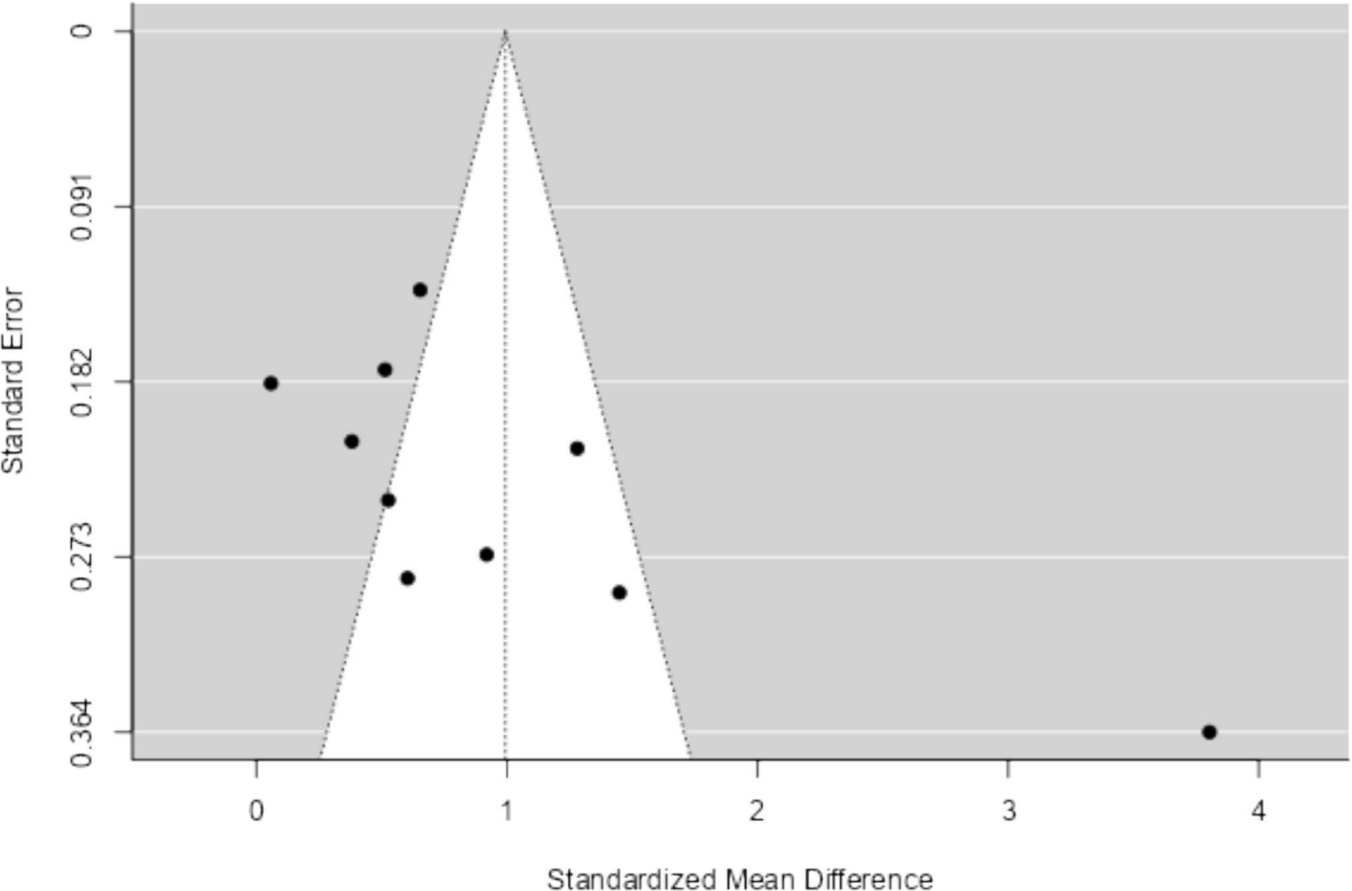
Funnel plot assessing publication bias in studies on EI intervention Effectiveness.

**Figure 5:**
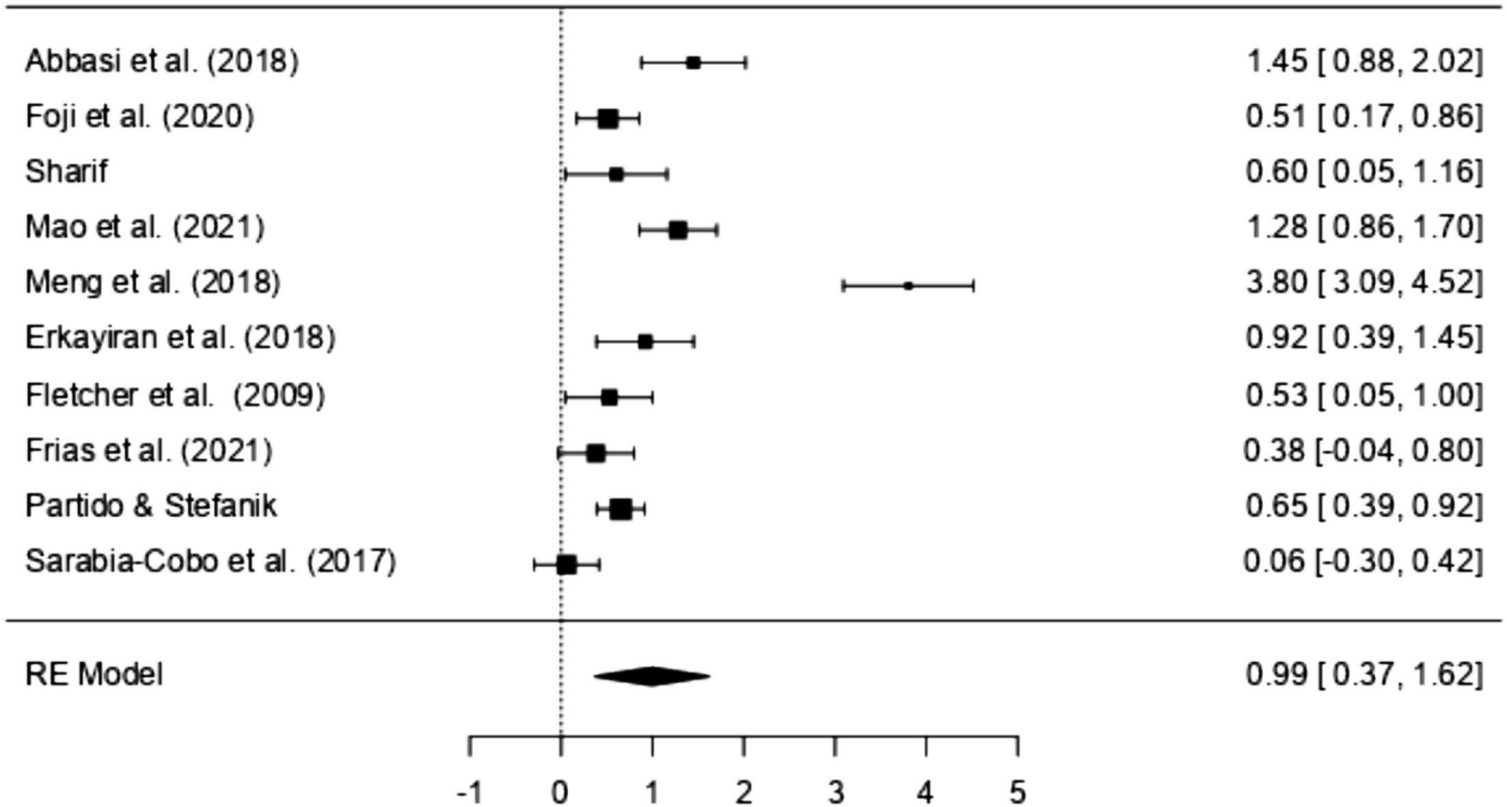
Combined forest plot of all studies assessing overall effectiveness of EI interventions.

The results of Funnel Plot Analyses examining the intervention effect on EI are presented in Figure 4. Following the recommendation by Higgins et al. (2011) for a minimum of ten studies to investigate small study effects or publication bias, all ten available studies were pooled together for this analysis. Both the visual inspection of the figure and Egger’s regression test indicated funnel plot asymmetry (*p* < 0.05), suggesting the presence of potential bias. Notably, one study (Meng and Qi, 2018) appeared to be particularly influential and emerged as an outlier in this analysis. The results of the overall Random Effects Model (*n* = 10) can be found in Table 6 and Figure 5.

**Table 6:** The overall results of the random effects model.

Effect size and 95% confidence interval				Prediction interval			Heterogeneity statistics					
Number of studies	Standard mean difference (SMD)	Standard error	Lower limit	Upper limit	Lower limit	Upper limit	Tau	Tau^2^	Q-value	df	*p*-value	I-square (%)
10	0.99	0.32	0.37	1.62	−1.03	3.02	0.98	0.96 (SE = 0.48)	103.46	9	<0.001	95.39%

Restricted maximum-likelihood is the selected Tau^2^ estimator.

## 4 Discussion

### 4.1 Main findings

The present systematic and meta-analysis reviewed 17 studies to examine the effectiveness of EI interventions as a trainable skill among healthcare workers. These studies exhibited noticeable variations in terms of intervention duration, the types of interventions applied, and methodologies. The duration of interventions ranged from brief two-hour training sessions (Frias et al., 2021) to more extensive training programs extending up to seven months (Fletcher et al., 2009). Moreover, various intervention methods were employed to bolster EI among healthcare professionals, including the use of educational tools (Abbasi et al., 2018; Foji et al., 2020; Sharif et al., 2013; Partido and Stefanik, 2020), persuasion techniques (Kozlowski et al., 2018), training methods (Zijlmans et al., 2015; Erkayiran and Demirkiran, 2018; Fletcher et al., 2009; Karimi et al., 2020; Zijlmans et al., 2011), and behavior change strategies (Frias et al., 2021). Some studies adopted a hybrid approach by combining two or more of these intervention types (Mao et al., 2021; Meng and Qi, 2018; Kozlowski et al., 2018; Tadmor et al., 2016; Ragab et al., 2021; Shahid et al., 2018; Sarabia-Cobo et al., 2017). Remarkably, with the exception of a single study, all reported a significant improvement in EI following the interventions. The effect size for EI enhancement in RCTs and quasi-experimental studies ranged from moderate to large, while the effectiveness of EI interventions appeared to decrease with non-RCT designs, yielding smaller effect sizes. This result supports previous findings confirming the effectiveness of interventions to increase EI (Mattingly and Kraiger, 2019; Hodzic et al., 2018; Kotsou et al., 2019). However, a major limitation across the reviewed studies was the scarcity of empirical data and flawed methodologies (including unconcealed participant allocation and poor randomisation), which contributed to a high risk of bias in many studies. Due to this publication bias, it can be assumed that the true effect of EI intervention across these methodologies is much smaller than reported. In terms of secondary measures, although not extensively explored in this review, the findings suggest potential benefits to the healthcare workforce following EI training. These benefits encompassed those for the individual including improved health, stress management, resilience and empowerment (Foji et al., 2020; Mao et al., 2021; Sharif et al., 2013; Karimi et al., 2020) and systemic benefits such as improved communication, problem-solving abilities, quality of care and transformative leadership skills (Mao et al., 2021; Meng and Qi, 2018; Karimi et al., 2020; Frias et al., 2021). The findings present valuable contributions to the current body of knowledge on EI and offer important insights and recommendations for shaping future intervention strategies.

Despite the noted limitations, these findings provide preliminary insights into the potential benefits of EI interventions. Notably, the included studies bring to light the diversity in the duration of interventions, with longer interventions (typically spanning over ten sessions across several months) being the predominant choice among the reviewed studies. These preliminary findings suggest that employing a longer duration may be conducive to more substantial EI development. For instance, studies that embraced lengthier interventions frequently reported more favourable outcomes in terms of EI enhancement [e.g., (Mao et al., 2021; Meng and Qi, 2018)]. Conversely, when the duration of the intervention was shorter, typically less than one month, it often resulted in less pronounced impacts on EI development [e.g., (Sharif et al., 2013; Sarabia-Cobo et al., 2017)]. This contrast is exemplified in the study conducted by Frias et al. (2021), which revealed a non-significant difference in EI following their brief two-hour intervention. They identified a limitation in their approach, notably the minimal interaction between researchers and participants during the four months of the intervention and post-survey, which may have contributed to the less substantial effects observed. This suggests that an essential component to enhance the effectiveness of these interventions requires a more gradual dissemination of information to sustain learning transfer and the acquisition of EI competencies. This might involve more detailed skill training and providing participants with additional time to become accustomed to thinking in more adaptive ways, which might not be achievable in a short two-hour intervention (Kotsou et al., 2019). Considering that EI is often considered trait-related, it is plausible that more extended durations, suggested to be between four to eight weeks, are required for the newly acquired EI skills to become internalised and automatic (Roberts et al., 2017). However, it is important to note that these conclusions may vary among scholars. Kruml and Yockey (2011) examined whether different modes of training delivery and training lengths (ranging from a 7-week to 16-week intervention) would lead to different outcomes and reported no significant differences in effectiveness among the different settings. Additionally, when comparing a long-term (13-week) with a short-term (2-days) coaching skills training program, Grant (2007) found that EI increased only with the long-term intervention. Therefore, these findings serve as a preliminary basis for further exploration of the optimum intervention length. Given the methodological variability and limited scope of high-quality studies in this field, future research is needed to establish the optimal duration and structure of EI interventions more reliably. One suggestion outlined by Roberts et al. (2017) is for researchers to consider longitudinal studies that assess changes on a more frequent basis, such as every week or month, to capture when and how these changes occur.

Beyond the varied intervention durations, the reviewed studies also displayed substantial diversity in their approaches and models for enhancing EI. For example, many of the included studies used didactic training [e.g., (Abbasi et al., 2018; Sharif et al., 2013)], while others used reflective or collaborative approaches [e.g., (Karimi et al., 2020)], or a combination of both [e.g., (Meng and Qi, 2018; Ragab et al., 2021; Sarabia-Cobo et al., 2017)]. While the exact reasons why the intervention was effective are beyond this review’s scope, previous literature has indicated that EI scores tend to improve when participants engage actively in practising and receive constructive feedback (Mattingly and Kraiger, 2019). An illustrative example of an effective EI intervention can be found in Mao et al. (2021) study, which employed a two-phase approach. The first phase encompassed formal lectures based on Mayer and Salovey’s abilities model of EI (comprising the abilities to perceive, use, understand and manage emotions), while the second phase focused on case management related to emotional control. During this phase, participants engaged in group discussions and received feedback from educators regarding their performance and strategies. The incorporation of both didactic and pedagogical approaches in this model might have contributed to its effectiveness in enhancing EI compared to other approaches reported in studies. The multifaceted nature of this training, designed to expand participants’ emotional knowledge and skills while increasing their emotional competence and confidence through practice, suggests that the combination of both approaches could be optimal. This aligns with prior literature endorsing the use of multiple methods for their potential to enhance EI (Mattingly and Kraiger, 2019; Hodzic et al., 2018; Geßler et al., 2021) and could serve as a valuable reference for those intending to develop future EI interventions.

An additional implication to highlight from this review pertains to the diversity of methodologies employed within the studies. They encompass a range of approaches, including RCT (Abbasi et al., 2018; Foji et al., 2020; Mao et al., 2021; Meng and Qi, 2018; Sharif et al., 2013; Zijlmans et al., 2015), quasi-experimental designs with pre-test-post-test control groups (Erkayiran and Demirkiran, 2018; Fletcher et al., 2009; Kozlowski et al., 2018), controlled before-after studies (Karimi et al., 2020; Tadmor et al., 2016; Zijlmans et al., 2011), and non-RCT (Frias et al., 2021; Partido and Stefanik, 2020; Ragab et al., 2021; Shahid et al., 2018; Sarabia-Cobo et al., 2017). From the meta-analytic, it can be inferred that RCTs and quasi-experimental designs were particularly useful in assessing the intervention’s effectiveness, as evidenced by the positive correlations found with EI. Random assignment, as well as quasi-experimental designs, have been extensively researched and are considered advantageous in the context of EI interventions, as they help control for motivation and demand effects, ultimately supporting the establishment of causal relationships and allowing rigorous empirical comparison of results (Mattingly and Kraiger, 2019; Hodzic et al., 2018). Therefore, it is recommended that future studies consider incorporating randomisation and active control groups to engage in similar activities during the EI intervention (e.g., a mindfulness or relaxation control group conducted for the same duration). However, given the notable publication bias reported within these studies, possibly explained by the poor methodological quality or non-reporting of results, it is essential to interpret these findings cautiously. This bias emphasises the need for future researchers to explore these varied methodological properties more comprehensively to gain a deeper understanding of their impact on intervention effectiveness.

### 4.2 Limitations and future research directions

While the findings of this review offer intriguing insights that could enhance our understanding of EI interventions for healthcare workers, several limitations should be acknowledged. A notable limitation of this review is the substantial variability in study characteristics among the included studies, particularly regarding intervention duration, types of interventions, and methodologies. Interventions ranged from brief two-hour training sessions to extensive programs lasting up to seven months, with differing approaches and evaluation methods. This heterogeneity may limit the comparability of study outcomes and the generalizability of our findings. While our analysis aimed to account for these variations, the diversity in intervention designs and durations makes it challenging to draw definitive conclusions about the overall effectiveness of interventions targeting healthcare workers. Future research would benefit from more standardized intervention protocols to facilitate clearer comparisons and more robust conclusions. Furthermore, the relatively small sample sizes across the included studies may have impacted the certainty of the results, and the actual effect could differ significantly from what was observed in this review, making real-world effects uncertain. Moreover, the predominant presence of female participants in the study sample aligns with the global representation of the healthcare workforce, where women constitute around 70% (Boniol et al., 2019). However, given the ongoing efforts to enhance gender diversity in the healthcare sector, future studies should consider larger and more gender-diverse samples.

Another limitation that may have resulted in an overestimation of the true effect concerns the consistent methodological flaws found in the reviewed studies. As mentioned earlier, several of the studies lacked crucial elements like randomisation and allocation concealment. Moreover, since all these studies relied on self-reported measures, concerns about potential biases and their impact on the true effects of the interventions arise. While it is possible that some studies did incorporate these vital elements, the scarcity of specific details within their documentation introduces an element of uncertainty. This uncertainty extends to basic statistical information, such as sample sizes, means, and standard deviations, which were often missing from the results section. This omission complicates the calculation of effect sizes. Additionally, many authors provided only vague descriptions of their intervention programs, lacking comprehensive information. Consequently, it is strongly recommended that future researchers furnish relevant data regarding their results and offer detailed descriptions of the treatment modalities. This not only eases the application of meta-analytic procedures but also advances our understanding of the effectiveness of EI interventions. Such knowledge facilitates the dissemination of effective interventions for this population and aids in better replication, assessment, and comprehension of the actual effects of EI interventions.

Furthermore, clear evidence of publication bias is discernible among these studies. Given that nearly all included studies reported a significant effect, it can be inferred that studies with substantial effects are more readily published. This phenomenon, which has also been reported in other studies evaluating EI interventions (Hodzic et al., 2018), suggests that effect sizes in additional unpublished works examining the impact of training on EI may deviate significantly from the effect sizes documented in peer-reviewed journals and dissertations/theses. Therefore, it is recommended that future reviews consider including dissertations and unpublished reports to access a more accurate representation of the true results.

Future research should also consider conducting a subgroup analysis comparing the outcomes of EI interventions assessed through self-report tools versus performance-based tools. Self-report measures and performance-based assessments capture different dimensions of EI, self-perceived abilities versus objective emotional processing skills, and comparing these tools could provide valuable insights into how each type reflects changes following interventions. Such a distinction would contribute to a more nuanced understanding of EI training efficacy in healthcare settings and beyond.

### 4.3 Conclusion

In conclusion, this review underscores the potential benefits of implementing EI interventions for healthcare workers. The moderate positive effect observed suggests that EI is a trainable skill, offering opportunities for improving EI-related outcomes. Notably, interventions of longer duration and those incorporating multiple methods appear to be more effective. However, it is essential to approach these findings cautiously due to the methodological limitations and publication bias prevalent in the reviewed studies. To advance the field, future research should prioritise robust methodologies, including RCTs and quasi-experimental designs, and should also aim for larger and more diverse samples to assess intervention effectiveness.

## Author’s note

This manuscript includes original work not previously published or presented and not under current review elsewhere. All data and syntax supporting the findings of this study are available with the article and its Supplementary materials. The present systematic and meta-analysis has been pre-registered with PROSPERO [CRD42023393760]. Further details about the registration can be accessed at: https://www.crd.york.ac.uk/prospero/display_record.php?ID=CRD42023393760.

## Data Availability

The original contributions presented in the study are included in the article/Supplementary material, further inquiries can be directed to the corresponding author.
