# Supplementary material for: Emotional intelligence training among the healthcare workforce: a systematic review and meta-analysis
Source: Front Psychol. 2024 Nov 20;15:1437035. doi: 10.3389/fpsyg.2024.1437035 (PMC11614651; doi:10.3389/fpsyg.2024.1437035)
Supplement: Supplementary file 1 [file Table_1.DOCX]

**Supplementary Document**

| **Supplementary Table 1**   \| Risk of assessment of included articles with support for judgement \| \| --- \| | | | | | | | |
| --- | --- | --- | --- | --- | --- | --- | --- | --- |
| First author and year | Selection bias | | Performance bias | Detection bias | Attrition bias | Reporting bias | Other bias |
| Fletcher et al. (2009) | ? | ? | - | - | - | ? | - |
|  | *Random sequence generation (selection bias)* has been marked as unsure due to the random allocation being based upon clinical rotation, with those who had a single clinical rotation being designated for the intervention group, while the control group were recruited from the other four clinical rotations. This allocation method may introduce an element of predictability.  *Allocation concealment (selection bias)* has been noted as unsure as there is a lack of information regarding the allocation concealment approach.  *Performance bias* was marked as high due to no personnel blinding being reported and as the participants were required to attend additional curriculum, therefore, it is assumed that they were not blinded.  *Detection bias* was marked as high as participants were the outcome assessors, however, given no participant blinding this can lead to bias in results.  *Attrition bias* was assessed as high due to the high rates of attrition (50%) with insufficient reporting for the reason why this occurred. Therefore, this may have impacted judgment and resulted in potential bias.  *Reporting bias* has been marked as unsure due to insufficient information provided regarding the pre-registration of actual outcomes or protocol.  *Other Bias* was assessed as high stemming from the scarcity of information provided. In particular, the study did not disclose whether it was pre-registered, and there was also a lack of a comprehensive description of the intervention employed. | | | | | | |
| Abbasi et al. (2018) | ? | ? | ? | - | ? | ? | - |
|  | *Random sequence generation (selection bias)* has been marked as unsure due to insufficient information provided. The authors did note that the sample was randomly selected from several medical schools, however, the procedure was not provided.  *Allocation concealment (selection bias)* has been noted as unsure as there is a lack of information regarding the allocation concealment approach.  *Performance bias* was marked as unsure due to insufficient information to permit judgment as there was no information regarding the blinding of participants and personnel.  *Detection bias* was marked as unsure due to insufficient detail provided regarding the blinding of the outcome assessment.  *Attrition bias* was appraised as high owing to inadequate reporting of data handling procedures. In particular, the absence of information regarding the rationale behind the reduction in the number of participants (initially from 120 to 67 participants) and the subsequent downsizing to 30 participants in each group contributed to this assessment.  *Reporting bias* has been marked as unsure due to insufficient information provided regarding the pre-registration of actual outcomes or protocol. However, all outcomes related to EI were reported.  *Other Bias* was assessed as high stemming from the scarcity of information provided. In particular, the study did not disclose whether it was pre-registered, and there was also a lack of a comprehensive description of the intervention employed. | | | | | | |
| Partido & Stefanik, 2020 | - | - | - | - | + | ? | ­- |
|  | *Random sequence generation (selection bias)* has been marked as high attributed to the absence of a randomized sequencing process and the use of a pre-posttest design.  *Allocation concealment (selection bias)* was assessed as high due to the lack of information on whether allocation sequence concealment measures were implemented, and the study's inherent high bias risk associated with its pre-post design without randomization or a control group.  *Performance bias* was marked as high due to the nature of the training/intervention where blinding is not possible.  *Detection bias* was assigned a high risk due to the inherent nature of the training/intervention, where blinding was not possible, compounded by the fact that EI was self-assessed by participants using questionnaires. Consequently, these circumstances could potentially have influenced the study's outcomes.  *Attrition bias* was appraised as low due to the low attrition rates (117/200).  *Reporting bias* has been marked as unsure due to insufficient information provided regarding the pre-registration of actual outcomes or protocol. However, all outcomes related to EI were reported.  *Other Bias* was assessed as high due to insufficient information provided, specifically, they did not note if the study was pre-registered. | | | | | | |
| Ragab et al. (2021) | - | - | - | - | + | ? | - |
|  | *Random sequence generation (selection bias)* has been marked as high attributed to the absence of a randomized sequencing process and the use of a pre-posttest design.  *Allocation concealment (selection bias)* was assessed as high due to the lack of information on whether allocation sequence concealment measures were implemented, and the study's inherent high bias risk associated with its pre-post design without randomization or a control group.  *Performance bias* was marked as high due to the nature of the training/intervention where blinding is not possible.  *Detection bias* was assigned a high risk due to the inherent nature of the training/intervention, where blinding was not possible, compounded by the fact that EI was self-assessed by participants using questionnaires. Consequently, these circumstances could potentially have influenced the study's outcomes.  *Attrition bias* was appraised as low due to the low attrition rates.  *Reporting bias* has been marked as unsure due to insufficient information provided regarding the pre-registration of actual outcomes or protocol. However, all outcomes related to EI were reported.  *Other Bias* was assessed as high due to insufficient information provided, specifically, they did not note if the study was pre-registered. | | | | | | |
| Meng 2018 | ? | ? | - | - | + | ? | - |
|  | *Random sequence generation (selection bias)* has been marked as unsure due to the insufficient information provided for the randomization procedure.  *Allocation concealment (selection bias)* has been noted as unsure as there is a lack of information regarding the allocation concealment approach.  *Performance bias* was deemed high because the control group lacked active conditions, increasing the likelihood that participants could discern which intervention they received. Furthermore, the absence of information about blinding added to this concern.  *Detection bias* was marked as high as participants were the outcome assessors, however, given no participant blinding this can lead to bias in results.  *Attrition bias* was assessed as low due to the low attrition rates.  *Reporting bias* has been marked as unsure due to insufficient information provided regarding the pre-registration of actual outcomes or protocol. However, all outcomes related to EI were reported.  *Other Bias* was assessed as unsure due to insufficient information provided, specifically, they did not note if the study was pre-registered. | | | | | | |
| Ramzan Shahid et al. (2018) | - | - | - | - | - | ? | - |
|  | *Random sequence generation (selection bias)* has been marked as high attributed to the absence of a randomized sequencing process and the use of a pre-posttest design.  *Allocation concealment (selection bias)* was assessed as high due to the lack of information on whether allocation sequence concealment measures were implemented, and the study's inherent high bias risk associated with its pre-post design without randomization or a control group.  *Performance bias* was marked as high due to the nature of the training/intervention where blinding is not possible.  *Detection bias* was assigned a high risk due to the inherent nature of the training/intervention, where blinding was not possible, compounded by the fact that EI was self-assessed by participants using questionnaires. Consequently, these circumstances could potentially have influenced the study's outcomes.  *Attrition bias* was deemed high due to substantial attrition rates (14 out of 45, equivalent to 35%) and the unavailability of participants for follow-up, primarily resulting from their graduation.  *Reporting bias* has been marked as unsure due to insufficient information provided regarding the pre-registration of actual outcomes or protocol. However, all outcomes related to EI were reported.  *Other Bias* was assessed as high due to insufficient information provided, specifically, they did not note if the study was pre-registered. | | | | | | |
| Erkayiran et al. (2018) | + | ? | ? | - | + | ? | - |
|  | *Random sequence generation (selection bias)* has been low due to the implementation of random allocation via random number generation.  *Allocation concealment (selection bias)* has been noted as unsure as there is a lack of information regarding the allocation concealment approach.  *Performance bias* was marked as unsure due to insufficient information to permit judgment as there was no information regarding the blinding of participants and personnel.  *Detection bias* was marked as high as participants were the outcome assessors, however, given there is insufficient information regarding the blinding of the control group this may have greatly impacted the findings.  *Attrition bias* was deemed low due to the lack of missing data presented in the tables and figures.  *Reporting bias* has been marked as unsure due to insufficient information provided regarding the pre-registration of actual outcomes or protocol. However, all outcomes related to EI were reported.  *Other Bias* was assessed as high due to insufficient information provided, specifically, they did not note if the study was pre-registered. | | | | | | |
| Foji et al. (2020) | + | ? | ? | - | + | ? | - |
|  | *Random sequence generation (selection bias)* has been low due to the implementation of random allocation via a random number table and list of sample group names.  *Allocation concealment (selection bias)* has been noted as unsure as there is a lack of information regarding the allocation concealment approach.  *Performance bias* was deemed unsure due to the control group lacking active conditions, increasing the likelihood that participants could discern which intervention they received. Furthermore, the absence of information about blinding added to this concern.  *Detection bias* was marked as high as participants were the outcome assessors, however, given there is insufficient information regarding the participant blinding this may have greatly impacted the findings.  *Attrition bias* was assessed as low due to the low attrition rates.  *Reporting bias* has been marked as unsure due to insufficient information provided regarding the pre-registration of actual outcomes or protocol. However, all outcomes related to EI were reported.  *Other Bias* was assessed as high due to insufficient information provided, specifically, they did not note if the study was pre-registered. | | | | | | |
| Zijlmans et al. (2015) | ? | - | ? | - | + | ? | - |
|  | *Random sequence generation (selection bias)* was categorized as unsure due to the absence of explicit information concerning the methodology used for randomizing the experimental and control groups. The author's statement that "Within these teams, staff members were selected randomly to participate in the experimental group. The remaining staff members of these teams participated in control group 1" left room for ambiguity, making it challenging to definitively evaluate the selection bias.  *Allocation concealment (selection bias)* was assessed as high due to the following statement “The first one and a half day consisted of in-service, didactic training sessions focused on the concept of EI and its significance for both profession and teamwork” which suggests that the allocation process was not adequately concealed, potentially leading to bias in participant assignment.  *Performance bias* was marked as unsure due to insufficient information to permit judgment as there was no information regarding the blinding of participants and personnel.  *Detection bias* was marked as high as participants were the outcome assessors, however, given there is insufficient information regarding the participant blinding this may have greatly impacted the findings.  *Attrition bias* was deemed low due to the low attrition rate reported for the experimental and control groups. Additionally, no exclusion criteria were reported.  *Reporting bias* has been marked as unsure due to insufficient information provided regarding the pre-registration of actual outcomes or protocol. However, all outcomes related to EI were reported.  *Other Bias* was assessed as high due to insufficient information provided, specifically, they did not note if the study was pre-registered. | | | | | | |
| Frias et al. (2021) | - | - | - | - | - | ? | - |
|  | *Random sequence generation (selection bias)* was categorized as high due to the absence of a randomized sequencing process and the utilization of a within-group design rather than a mixed design, potentially introducing bias into the study.  *Allocation concealment (selection bias)* was assessed as high due to the lack of information on whether allocation sequence concealment measures were implemented, and the study's inherent high bias risk associated with a within-group design without randomization or a control group.  *Performance bias* was marked as high due to the nature of the training/intervention where blinding is not possible.  *Detection bias* was assigned a high risk due to the inherent nature of the training/intervention, where blinding was not possible, compounded by the fact that EI was self-assessed by participants using questionnaires. Consequently, these circumstances could potentially have influenced the study's outcomes.  *Attrition bias* was deemed high due to substantial attrition rates (18 out of 43, equivalent to ~60%) that were not explained in the study.  *Reporting bias* has been marked as unsure due to insufficient information provided regarding the pre-registration of actual outcomes or protocol. However, all outcomes related to EI were reported.  *Other Bias* was assessed as high due to insufficient information provided, specifically, they did not note if the study was pre-registered. | | | | | | |
| Zijlmans et al. (2011) | - | - | ? | - | + | ? | - |
|  | *Random sequence generation (selection bias)* was categorized as high due to the absence of randomized sequencing.  *Allocation concealment (selection bias)* was appraised as high due to the inherent nature of the intervention, which necessitated feedback both orally and through video for each participant's EQ-I profile, suggesting that allocation concealment was not possible within the study design.  *Performance bias* was marked as unsure due to insufficient information to permit judgment as there was no information regarding the blinding of participants and personnel.  *Detection bias* was assigned a high risk due to the inherent nature of the training/intervention, where blinding was not possible, compounded by the fact that EI was self-assessed by participants using questionnaires. Consequently, these circumstances could potentially have influenced the study's outcomes.  *Attrition bias* was considered low because while attrition was observed in the sample, an independent t-test did not reveal any significant differences between those participants who completed the intervention and those who dropped out, suggesting that attrition was unlikely to have substantially biased the study's outcomes.  *Reporting bias* has been marked as unsure due to insufficient information provided regarding the pre-registration of actual outcomes or protocol. However, all outcomes related to EI were reported.  *Other Bias* was assessed as high due to insufficient information provided, specifically, they did not note if the study was pre-registered. | | | | | | |
| Tadmor et al. (2016) | - | - | - | ? | + | ? | - |
|  | *Random sequence generation (selection bias)* has been marked as high attributed to the absence of a randomized sequencing process and the use of a pre-posttest design.  *Allocation concealment (selection bias)* was assessed as high due to the lack of information on whether allocation sequence concealment measures were implemented, and the study's inherent high bias risk associated with its pre-post design without randomization or a control group.  *Performance bias* was marked as high due to the nature of the training/intervention where blinding is not possible. Moreover, given the training program explicitly about EI, no blinding of personnel was possible.  *Detection bias* was assigned as an unsure risk due to the feedback session occurring after the post-training measurement took place, suggesting that it is unlikely that the sample was blinded by the outcome assessors. Additionally, EI was self-assessed by participants using questionnaires.  *Attrition bias* was deemed low due to the low attrition rate reported for the experimental and control groups.  *Reporting bias* has been marked as unsure due to insufficient information provided regarding the pre-registration of actual outcomes or protocol. However, all outcomes related to EI were reported.  *Other Bias* was assessed as high due to insufficient information provided, specifically, they did not note if the study was pre-registered. Additionally, there was a conflict of interest present with two authors indicating financial interest in the group that led the intervention. | | | | | | |
| Mao et al. (2021) | + | ? | ? | - | + | ? | - |
|  | *Random sequence generation (selection bias)* has been low due to the implementation of random allocation via a web-based random number generator.  *Allocation concealment (selection bias)* has been noted as unsure as there is a lack of information regarding the allocation concealment approach.  *Performance bias* assessment remains uncertain due to the lack of detailed information regarding blinding procedures and the study's conditions, which implies that blinding of both participants and personnel might not have been feasible. Although both the experimental and control groups received some form of active treatment (i.e., daily departmental training without EI components), the participants' potential interactions with each other within the same hospital raise the possibility of information exchange. The absence of explicit blinding protocol information prevents any conclusive judgment in this regard.  *Detection bias* was assigned a high risk due to the inherent nature of the training/intervention, where blinding was not possible, compounded by the fact that EI was self-assessed by participants using questionnaires. Consequently, these circumstances could potentially have influenced the study's outcomes.  *Attrition bias* was assessed as low due to the low attrition rates reported (i.e., the intervention group lost 18% (from 53 to 43), and the control group lost 10% (50 to 45)). All outcomes were reported.  *Reporting bias* has been marked as unsure due to insufficient information provided regarding the pre-registration of actual outcomes or protocol. However, all outcomes related to EI were reported.  *Other Bias* was assessed as high due to insufficient information provided, specifically, they did not note if the study was pre-registered. | | | | | | |
| Sharif et al. (2013) | ? | ? | - | ? | + | + | + |
|  | *Random sequence generation (selection bias)* has been marked as unsure due to the insufficient information provided for the randomization procedure.  *Allocation concealment (selection bias)* has been noted as unsure as there is a lack of information regarding the allocation concealment approach.  *Performance bias* was deemed high because the control group lacked active conditions, increasing the likelihood that participants could discern which intervention they received. Furthermore, the absence of information about blinding added to this concern.  *Detection bias* was marked as unsure due to insufficient detail provided regarding the blinding of the outcome assessment. Given the participants were the outcome assessors, this may have impacted the results.  *Attrition bias* was assessed as low due to the low attrition rates.  *Reporting bias* has been reported as low since the clinical trial was pre-registered, and there were no observable signs or indications of selective reporting within the text.  *Other Bias* was similarly appraised as low because no apparent additional risks were identified, and the study had been pre-registered. | | | | | | |
| Karimi et al. (2020) | - | ? | ? | - | - | ? | - |
|  | *Random sequence generation (selection bias)* has been marked as high attributed to the absence of a randomized sequencing process and the use of a quasi-experimental trial design.  *Allocation concealment (selection bias)* has been noted as unsure as there is a lack of information regarding the allocation concealment approach.  *Performance bias* was labelled as unsure due to insufficient information to permit judgment as there was no information regarding the blinding of participants and personnel. While the control group was advised that the study aimed to examine their work experience and the two groups were geographically distant from each other, there is still a possibility that participants were aware of which intervention they were part of due to the missing details on blinding procedures.  *Detection bias* was marked as high as participants were the outcome assessors, however, given there is insufficient information regarding the participant blinding this may have greatly impacted the findings.  *Attrition bias* was deemed high due to substantial attrition rates (~ 50%) that were not explained in the study.  *Reporting bias* has been marked as unsure due to insufficient information provided regarding the pre-registration of actual outcomes or protocol. However, all outcomes related to EI were reported.  *Other Bias* was assessed as high due to insufficient information provided, specifically, they did not note if the study was pre-registered. | | | | | | |
| Kozlowski et al. (2018) | - | ? | ? | - | ? | ? | - |
|  | *Random sequence generation (selection bias)* was categorized as high due to the absence of randomized sequencing.  *Allocation concealment (selection bias)* has been noted as unsure as there is a lack of information regarding the allocation concealment approach.  *Performance bias* was marked as unsure due to insufficient information to permit judgment as there was no information regarding the blinding of participants and personnel. The author did note that intervention and control groups were across sites and did not interact, however, there is still a possibility that the participants were aware of the intervention they received.  *Detection bias* was marked as high as participants were the outcome assessors.  *Attrition bias* was assessed as high due to the high rates of attrition (39/60 ~ 65%) with insufficient reporting for the reason why this occurred. Therefore, this may have impacted judgment and resulted in potential bias.  *Reporting bias* has been marked as unsure due to insufficient information provided regarding the pre-registration of actual outcomes or protocol. However, all outcomes related to EI were reported.  *Other Bias* was assessed as high due to insufficient information provided, specifically, they did not note if the study was pre-registered. | | | | | | |
| Sarabia-Cobo et al. (2017) | - | - | - | - | + | ? | - |
|  | *Random sequence generation (selection bias)* has been marked as high attributed to the absence of a randomized sequencing process and the use of a pre-posttest design.  *Allocation concealment (selection bias)* was assessed as high due to the lack of information on whether allocation sequence concealment measures were implemented, and the study's inherent high bias risk associated with its pre-post design without randomization or a control group.  *Performance bias* was marked as high due to the nature of the training/intervention where blinding is not possible.  *Detection bias* was assigned a high risk due to the inherent nature of the training/intervention, where blinding was not possible, compounded by the fact that EI was self-assessed by participants using questionnaires. Consequently, these circumstances could potentially have influenced the study's outcomes.  *Attrition bias* was appraised as low due to the low attrition rates at the one-year follow-up assessment (87/92).  *Reporting bias* has been marked as unsure due to insufficient information provided regarding the pre-registration of actual outcomes or protocol. However, all outcomes related to EI were reported.  *Other Bias* was assessed as high due to insufficient information provided, specifically, they did not note if the study was pre-registered. | | | | | | |
|  | Selection bias | | Performance bias | Detection bias | Attrition bias | Reporting bias | Other bias |
|  | Sequence generation | Allocation concealment | Blinding of participants and personnel | Blinding of outcome assessment | Incomplete outcome data | Selective reporting | Other sources of bias |
| *Note.*  + low risk of bias  - high risk of bias  ? unclear risk of bias | | | | | | | |
